# Study of crystallization mechanism of Al-based amorphous alloys by in-situ high temperature X-ray diffraction method

**DOI:** 10.1038/s41598-022-09640-9

**Published:** 2022-04-06

**Authors:** Rafał Babilas, Katarzyna Młynarek-Żak, Wojciech Łoński, Dariusz Łukowiec, Tymon Warski, Adrian Radoń

**Affiliations:** 1 Department of Engineering Materials and Biomaterials, Silesian University of Technology, Konarskiego 18a St, 44-100 Gliwice, Poland; 2 Łukasiewicz Research Network, Institute of Non-Ferrous Metals, Sowińskiego 5 St, 44-100 Gliwice, Poland

**Keywords:** Structural materials, Techniques and instrumentation

## Abstract

The role of transition metals (TMs) addition on the formation and crystallization of amorphous Al_85_TMs_10_Y_5_ alloys was described using in-situ high-temperature X-ray diffraction. The structural results were compared with differential scanning calorimetry and dynamical mechanical analysis to obtain detailed information about the nucleation and growth of crystalline phases. The performed analysis confirmed that Fe and Cu addition drastically changes the crystallization temperature and the phase composition of the fully crystallized alloys. While for Al_85_Ni_10_Y_5_ alloy, the second crystallization step is related to the formation of Al_19_Ni_5_Y_3_ phase, for Al_85_(Ni, Fe)_10_Y_5_ and Al_85_(Ni, Fe, Cu)_10_Y_5_ alloys crystallization of Al_15_Fe_9_Y_2_ phase was observed. Interestingly, the performed analysis showed that forming a homogenous amorphous phase is not necessary to obtain the best corrosion resistance. It was noted that the precipitation of the YCr_2_Al_20_ phase in the Cu-rich amorphous matrix should be a much more interesting approach.

## Introduction

Due to their high mechanical properties and corrosion resistance, aluminum-based amorphous and nanocrystalline alloys are widely studied in the literature^1–4^. Different alloying systems are very well known and tested in the context of the formation of pure amorphous alloys and amorphous-nanocrystalline alloys^5,6^. The possibility of amorphous phase formation in Al-based alloys was first confirmed for a few Al-based binary alloys containing, for example, Cu, Cr and Pd^7–9^. However, pure, homogenous amorphous alloys were obtained firstly for Al–Fe–B, Al–Co–B and Al–Fe–Si ternary systems^10,11^. Unfortunately, these alloys are very brittle. The alloys characterized by ductility and high tensile strength were obtained for Al–Y–Ni ternary system^12^. Based on these results, new alloying systems were proposed and tested in the context of the production of Al-based amorphous alloys with high mechanical properties^13–15^. Generally, presented in the literature, amorphous alloys belong to one of the three alloying systems: Al-RE, Al-ETM-LTM or Al-LTM-RE, where RE is the rare earth element, ETM and LTM are the early and late transition metals, respectively. In the case of alloys containing rare earth elements, it was shown that the different RE elements have a similar influence on the glass-forming ability (GFA), while the transition metals (TMs) play a crucial role in the formation and stability of amorphous phase^16^. For example, Rusanov et al. has shown that the simultaneous addition of 4 at.% Ni and 4 at.% Co improves the thermal stability of Al–Ni–Co–Yb amorphous metallic ribbons^17^. Also, Fe:Y concentration ratio in Al–Y–Fe alloys plays a crucial role in the glass-forming ability, which was presented by Babilas et al.^4^. The comprehensive research on the mechanical properties of Al-based amorphous alloys concluded that these alloys are unfortunately characterized by low thermal stability and, generally, by poor ductility. Accordingly, the recent works focused on preparing amorphous-nanocrystalline alloys, which combines unique properties of these two phases^18^.

A few different approaches, such as partial crystallization of amorphous precursors, physical vapor deposition, and grain boundary amorphization, were proposed and described in the literature to obtain amorphous-nanocrystalline alloys^18^. Kim et al.^19^ described the possibility of in-situ formation of intermetallic compounds during spark-plasma sintering of Al_84_Ni_7_Co_3_Dy_6_ amorphous alloy, which increases the compressive yield strength to 1433 MPa and maximum strength to 1773 MPa. When partial crystallization is one of the common ways to prepare amorphous-nanocrystalline alloys, the possibility of the control of this process is limited, which is strongly related to the lack of knowledge about the crystallization from an amorphous phase in the function of time and temperature^20^. One of the most using techniques, which can provide information about the crystallization process from the amorphous state, is the differential scanning calorimetry (DSC) and in-situ crystallization monitoring using transmission electron microscopy (TEM)^21^. The DSC can provide information about the number of crystallized phases and temperatures related to crystallite formation and growth. However, the shape and onset temperature are strongly heating rate dependent^22^. On the other hand, the analysis of TEM micrographs can provide more helpful information about the formation of crystallites from the amorphous phase, however, only locally and for ultrathin samples.

While the mechanical properties of crystalline, nanocrystalline, amorphous and amorphous-nanocrystalline Al-based alloys are widely studied, the influence of the chemical composition and structure on the corrosion of these alloys is still not well described in the literature. Ultimately, such materials are used as exposed elements to weathering, especially in shipbuilding and offshore structures. Accordingly, the corrosion behavior of amorphous and amorphous-crystalline alloys should also be studied and, above that, improved (for example, by the chemical or structural changes) like for their crystalline counterparts^23–25^.

Herein, the possibility of amorphous alloys formation in the Al_85_TMs_10_Y_5_ alloying system was tested for samples in the form of ribbons. Furthermore, the role of transition metals addition on glass-forming ability, corrosion resistance, and crystallization mechanism was studied in detail. According to our knowledge, this is the first study in which the *in-situ* high-temperature X-ray diffraction method was used to describe the crystallization mechanism in Al-based amorphous alloys in temperature and time domains. Moreover, obtained results were compared with thermal analysis to confirm the obtained results.

## Materials and methods

The chemical composition of Al_85_TMs_10_Y_5_ alloys was determined using the configurational entropy maximization approach. It is well known that the configurational entropy can reach maximum in alloys, in which the concentration of different chemical elements is the same^26^. According to that, the atomic concentration of Ni, Fe, Cu, and Cr was equal for all tested compositions. Therefore, for chosen to the analysis compositions (Al_85_Ni_10_Y_5_, Al_85_(Ni, Fe)_10_Y_5_, Al_85_(Ni, Fe, Cu)_10_Y_5_, Al_85_(Ni, Fe, Cr)_10_Y_5_ and Al_85_(Ni, Fe, Cu, Cr)_10_Y_5_) the entropy in the liquid state should be as high as possible and guarantee the random orientation of TMs dissolved in Al matrix. The samples in the form of ribbons were prepared from homogenous master alloys using melt spinning technique with constant wheel surface speed equal to 30 m/s. The differential thermal analysis (DTA) curves were measured for master alloys using NETSCH Jupiter STA 449 F3 thermal analyzer in a protective argon atmosphere with a constant heating/cooling rate equal to 20 K/min. The X-ray diffraction (XRD) patterns of casted alloys in the form of ribbons and in-situ high-temperature XRD patterns were measured using Rigaku MiniFlex 600 X-ray diffractometer equipped with the BTS 500 high-temperature attachment. The same samples were heated up to a specific temperature and held at this temperature for 60 min.

The XRD patterns were recorded with a constant time step Δ*t* equal to 15 min. Phase identification was performed using a dedicated Rigaku PDXL software suite. Verifying the amorphous state of the Al_85_TMs_10_Y_5_ alloys was performed by analyzing transmission electron microscopy micrographs and selected area electron diffraction (SAED) patterns collected using S/TEM TITAN 80–300. DSC curves were recorded using Netzsch DSC 404C Pegasus thermal analyzer at a 5 K/min heating rate. Dynamical mechanical analysis (DMA) was performed using Netzsch DMA 242E Artemis. The storage (*E′*) and loss (*E″*) modulus as a function of temperature were measured for amorphous alloys at a frequency equal to 1 Hz, with a heating rate equal to 2 K/min and under Ar atmosphere. Polarization curves and variation of the open-circuit potential with time were measured using a typical three-electrode cell using a sample as a working electrode, a saturated calomel electrode (SCE) as a reference electrode, and a platinum counter electrode.

## Results and discussion

**Figure 1:**
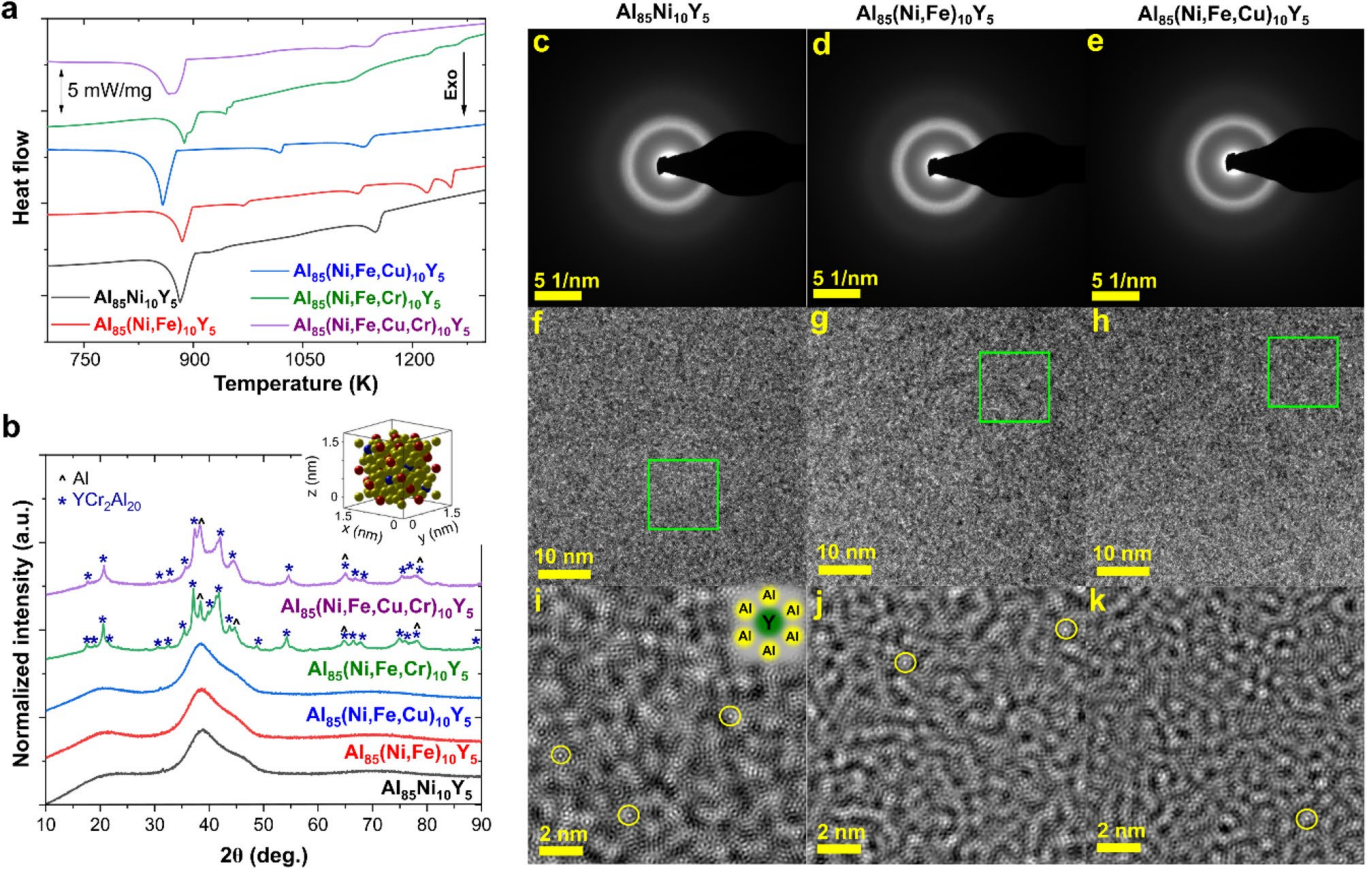
(**a**) DTA curves collected for crystalline alloys; (**b**) XRD patterns of amorphous and amorphous-crystalline ribbons with marked peaks corresponding to the Al and YCr_2_Al_20_ phases (insets shows the unit cell of the YCr_2_Al_20_ phase—Y, Cr, and Al were marked as blue, red and yellow balls, respectively); SAED patterns of Al_85_Ni_10_Y_5_ (**c**), Al_85_(Ni, Fe)_10_Y_5_ (**d**), and Al_85_(Ni, Fe, Cu)_10_Y_5_ (**e**) alloys with characteristic for amorphous phase halo ring pattern; HRTEM images with marked areas, which were enlarged to visualize atoms ordering in amorphous alloys for Al_85_Ni_10_Y_5_ (**f** and **i**), Al_85_(Ni, Fe)_10_Y_5_ (**g** and **j**), and Al_85_(Ni, Fe, Cu)_10_Y_5_ (**h** and **k**)—inset in (**i**) represent the identified atomic clusters, which were marked using yellow circles in figures (**i, j**).

The casting temperature was estimated based on DTA curves (Fig. 1a). All alloys are characterized by at least two exothermic processes related to the phase transformations. Despite that, the alloys contain high aluminum content (85 at.%) the casting temperature must be at least equal to 1350 K to obtain a homogenous liquid state. For samples in the form of ribbons, XRD patterns are shown in Fig. 1b. As one can see, forming a fully amorphous structure was possible for three alloys: Al_85_Ni_10_Y_5_, Al_85_(Ni, Fe)_10_Y_5_, and Al_85_(Ni, Fe Cu)_10_Y_5_. For alloys containing chromium, Al and YCr_2_A_l20_ phases were confirmed. Therefore, the addition of Cr leads to a reduction of the GFA in the Al_85_TMs_10_Y_5_ alloying system. To confirm the amorphous state of the Al_85_Ni_10_Y_5_, Al_85_(Ni, Fe)_10_Y_5_, and Al_85_(Ni, Fe, Cu)_10_Y_5_ alloys, the analysis of the TEM micrographs and SAED patterns was performed and presented in Fig. 1c–k. Both SAED patterns and TEM micrographs confirm the existence of the pure amorphous phase.

Moreover, the performed analysis confirms the existence of the characteristic for Al-RE and Al-RE-TM amorphous alloys atomic clusters (visualized in Fig. 1i and marked using yellow circles in Fig. 1i–k), in which the Y atom is surrounded by six aluminum atoms^27^. Although the highest entropy can be obtained for the alloy containing four different TMs, the possibility of amorphous structure formation is impossible using the proposed melt spinning method. The crucial role in this process plays a formation of the YCr_2_Al_20_ phase under rapid cooling.

**Figure 2:**
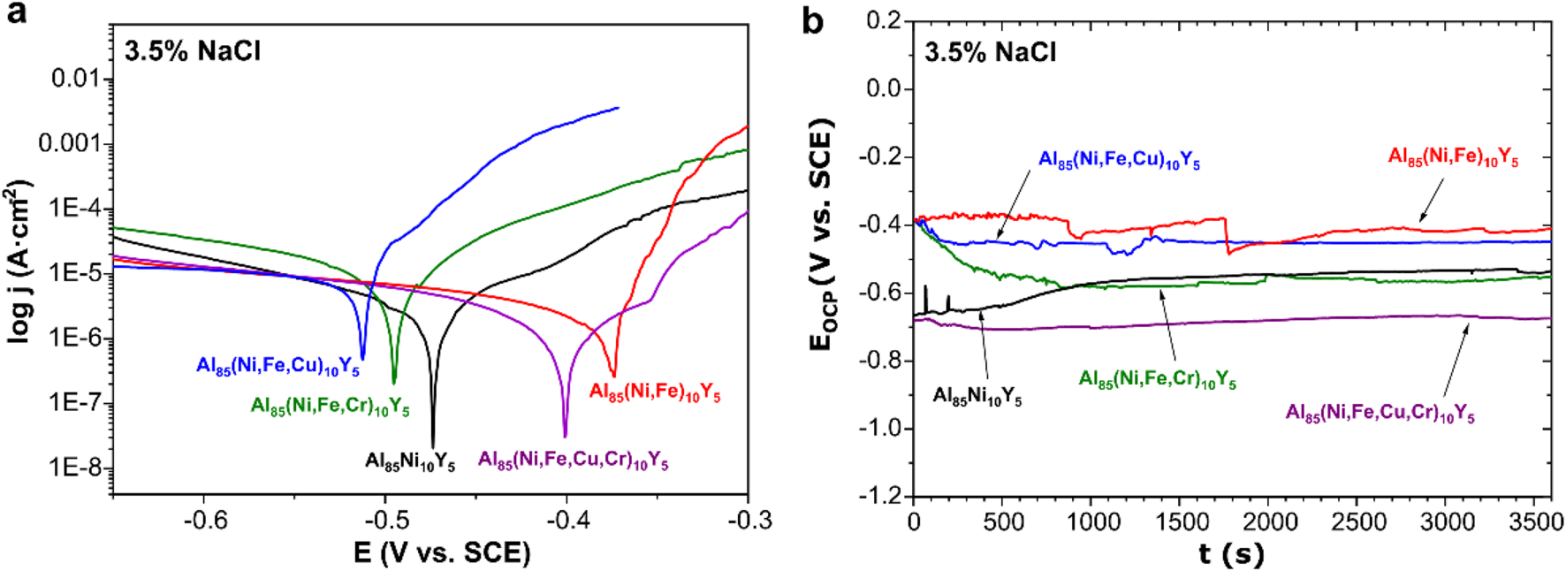
(**a**) Polarization curves and (**b**) Variation of the open-circuit potential with time measured for Al_85_TMs_10_Y_5_ alloys in 3.5% NaCl solution at 25 °C.

The formation of this intermetallic phase prevents the formation of fully amorphous alloys. However, CeCr_2_Al_20_-like phases, such as YCr_2_Al_20_, YbCr_2_Al_20_, and PrCr_2_Al_20,_ can enhance the precipitation-hardening behavior of Al-based alloys^28^. Moreover, the presence of these crystallites should also affect the other properties. According to that, the corrosion resistance was measured for all casted alloys to determine their further applicability. The obtained three fully amorphous and two partially crystalline alloys with Al and YCr_2_Al_20_ crystallites were tested. Polarization curves and variation of the open-circuit potential with time are presented in Fig. 2. Additionally, the values of open-circuit potential (*E*_OCP_), corrosion potential (*E*_corr_), polarization resistance (*R*_p_), and corrosion current density (*j*_corr_) determined by the Tafel and Stern-Geary method are summarized in Table 1.

**Table 1:** Results of polarization tests of Al_85_TMs_10_Y_5_ alloys in 3.5% NaCl solution at 25 °C.

Alloy	*E*_OCP_ [V]	*E*_corr_ [V]	*R*_*p*_ [kΩ cm^2^]	*j*_*corr*_ [μA/cm^2^]
Al_85_Ni_10_Y_5_	− 0.535	− 0.475	5.38	3.85
Al_85_(Ni, Fe)_10_Y_5_	− 0.411	− 0.376	3.73	2.12
Al_85_(Ni, Fe, Cr)_10_Y_5_	− 0.551	− 0.494	3.02	1.15
Al_85_(Ni, Fe, Cu)_10_Y_5_	− 0.673	− 0.512	1.13	6.28
Al_85_(Ni, Fe, Cu, Cr)_10_Y_5_	− 0.449	− 0.402	11.71	2.08

The worst corrosion resistance had the Al_85_(Ni, Fe, Cu)_10_Y_5_ amorphous alloy, while some of the more favorable values were determined for amorphous Al_85_(Ni, Fe)_10_Y_5_ and amorphous-crystalline Al_85_(Ni, Fe, Cu, Cr)_10_Y_5_ alloys. The literature data indicate that amorphous alloys are characterized by good corrosion resistance^29,30^. However, except for the structure, the chemical composition is one of the factors responsible for the material’s behavior in an electrochemical environment. According to that, the balance between structure and properties must be obtained. In the case of melt-spun ribbons, the obtained values of Al_85_(Ni, Fe, Cu, Cr)_10_Y_5_ alloy with crystallites indicate that the simultaneous addition of copper and chromium improves the corrosion resistance, which was also confirmed in the literature^31,32^. The addition of iron also positively affected the corrosion behavior compared to the Al_85_Ni_10_Y_5_ amorphous alloy. The positive effect of Fe was also observed in Al-Y alloys in the form of melt-spun ribbons for the Al_88_Y_7_Fe_5_ sample^4^. In the case of the addition of copper in the Al_85_(Ni, Fe, Cu)_10_Y_5_ alloy, the corrosion resistance was the worst, which is related to the high difference between the electrochemical potential of copper and aluminum.

**Figure 3:**
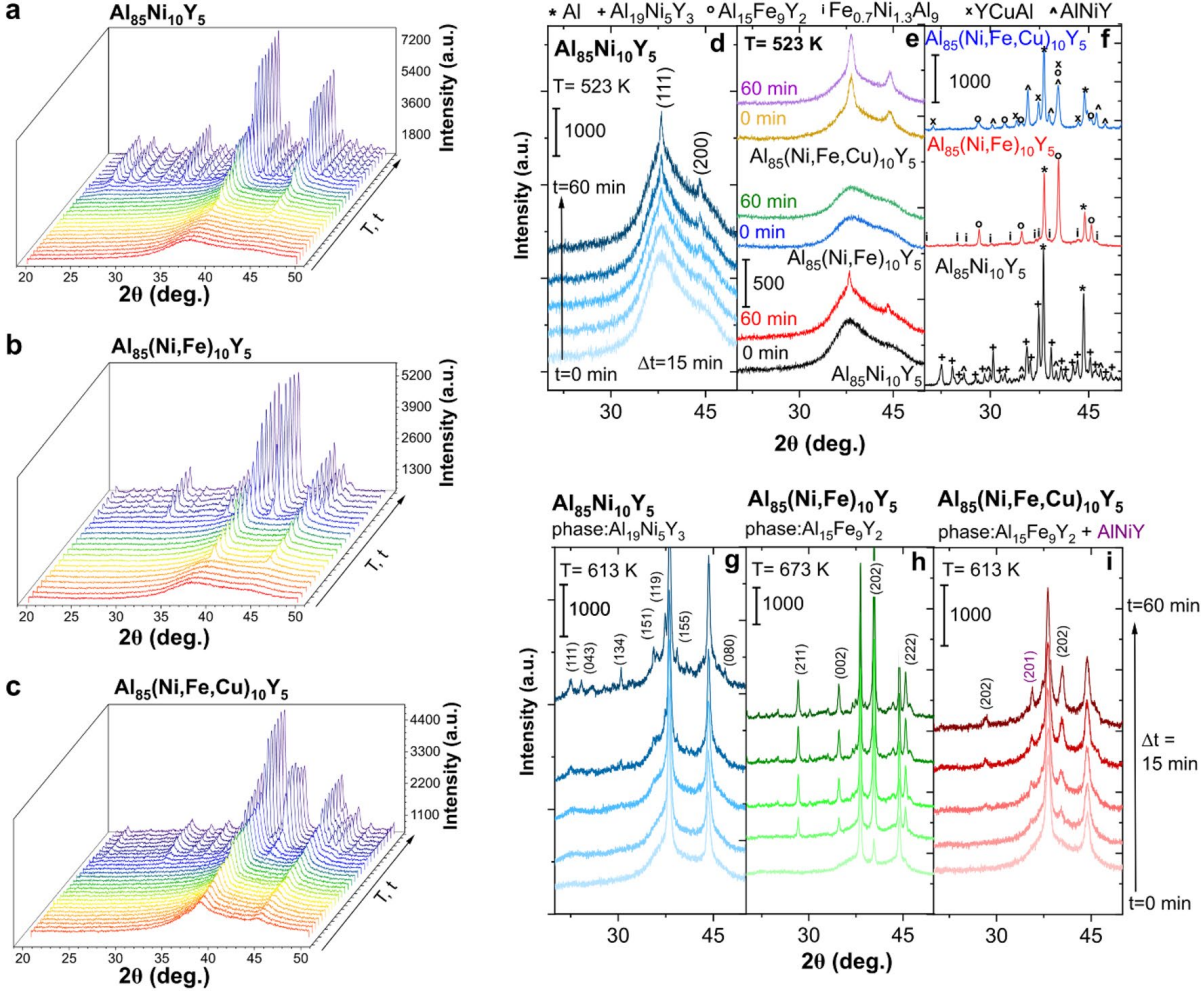
In-situ XRD patterns in temperature and time-domain recorded for (**a**) Al_85_Ni_10_Y_5_, (**b**) Al_85_(Ni, Fe)_10_Y_5_, and (**c**) Al_85_(Ni, Fe, Cu)_10_Y_5_ amorphous alloys; (**d**) time-dependent crystallization of Al phase presented in in-situ XRD patterns of Al_85_Ni_10_Y_5_ amorphous alloy at a constant temperature equal to 523 K; (**e**) comparison of the thermal stability of amorphous phase at a constant temperature equal to 523 K for 0 and 60 min of heating; (**f**) phase composition of fully crystallized amorphous alloys (the markings explained above the graphics); (**g**) crystallization of the second (Al_19_Ni_5_Y_3_) phase in Al_85_Ni_10_Y_5_ visualized in the time domain at T = 613 K; (**h**) crystallization of the second (Al_15_Fe_9_Y_2_) phase in Al_85_(Ni, Fe)_10_Y_5_ visualized in the time domain at T = 673 K; (**i**) spontaneous crystallization of the second (Al_15_Fe_9_Y_2_) and third (AlNiY) phases in Al_85_(Ni, Fe, Cu)_10_Y_5_ visualized in the time domain at T = 613 K;

While the knowledge about the role of chemical elements in amorphous and partially crystallized alloys can be used to casting the samples with increased corrosion resistance, the information about crystallization mechanism can provide helpful information to the preparation of amorphous-nanocrystalline alloys with the controlled size of crystallites and improved mechanical properties. Crystallization from the liquid and amorphous state must be treated as two different processes. In the liquid state, the mobility of all chemical elements is very high, whereas, in the amorphous system, their movements are prolonged. Therefore, the crystallization process from an amorphous, metastable state was described for the Al_85_TMs_10_Y_5_ alloying system. In different studies, information about the crystallization of different alloys was determined based on DSC analysis combined with the X-ray diffraction (XRD) patterns collected for isothermal annealing samples. Sometimes, these studies are supported by analysis of TEM images. In these studies, crystallization in time and temperature domains was analyzed using the XRD method, whereas patterns were collected *in-situ* under an annealing sample in certain conditions. A comprehensive crystallization mechanism was determined based on recorded XRD patterns. Firstly, the evolution of XRD patterns in the time and temperature domain, where the crystallization process can be easily observed, is presented in Fig. 3a–c. As can be seen, this process is different for all analyzed alloys—the substitution of Ni by Fe and Cu influences anticorrosion properties and the crystallization from the amorphous state. However, the first crystallization step is the same and is related to the formation and growth of the Al phase. This primary crystallization results in forming a metastable structure, in which Al nanocrystals coexist with the amorphous matrix and has been observed for other Al-based amorphous alloys such as Al_88_Y_7_Fe_5_ and Al_89_La_6_Ni_5_^33,34^. Moreover, this 1st stage can occur in the different temperature ranges for alloys containing different TMs. Analysis of crystallization of this phase in the time domain at a constant temperature equal to 523 K is presented in Fig. 3d for Al_85_Ni_10_Y_5_ alloy. As one can see, the intensity of two planes (111) and (200) increases simultaneously with progressive crystallization. The same situation was observed for other alloys. However, the temperature needed to start this process is different (Fig. 3e). While for Al_85_Ni_10_Y_5_ alloy, the crystallization of the Al phase starts at 523 K, and when the annealing time is equal to 30 min, the formation of Al crystallites cannot be observed even in the same temperature at 60 min for Al_85_(Ni, Fe)_10_Y_5_. On the other hand, the annealing of Al_85_(Ni, Fe, Cu)_10_Y_5_ amorphous alloy up to 523 K resulted in the formation of a well-crystallized Al phase. A similar finding of the role of Cu in crystallization of Al-based amorphous alloys have been presented by Kelhar et al. for Al_20.5_Ce_41.5_(Fe_y_Cu_1−y_)_38_ alloys^35^. The addition of Cu results in forming a fully amorphous structure in a wide Cu range (0 ≤ y ≤ 0.74), however, the Cu-rich alloys are characterized by lower crystallization temperature than alloys with higher Fe content.

Generally, the information about the Al nanocrystals precipitation in the amorphous matrix can be found in the literature for various Al-based amorphous alloys. However, the description of the crystallization of the other phases is complex and is usually presented as a phase analysis of a fully crystallized sample^34^. Sometimes, the crystallization mechanism can be described based on the XRD patterns collected for alloys isothermal annealed in different temperatures. For example, Svec et al.^36^, based on this approach, proposed a transformation sequence for Al_86_Ni_6_Co_2_Gd_6_ alloy. Unfortunately, such an analysis does not enable precise monitoring of the crystallization of individual phases. Accordingly, the in-situ XRD patterns analysis should be a good approach to describe the crystallization mechanism based on changes in the alloy in real-time, not only on the phase composition obtained after isothermal crystallization. As it was assumed, the most visible changes were observed for the crystallization of the 2nd and 3rd phases. The phase composition of fully crystallized alloys is slightly different, as seen in XRD patterns presented in Fig. 3f. When the alloy contains only Ni, the crystallization results in Al, Al_19_Ni_5_Y_3_, and AlNiY phases formatiom. Interestingly, the phase composition is different than for alloys containing higher yttrium content^37^; however, the presence of Al_19_Ni_5_Y_3_ phase was confirmed recently for similar crystallized Al_85_Ni_11_Y_4_ amorphous alloy^38^. Substitution of Ni by Fe changes the temperature of Al phase formation and the phase composition of the crystalline alloy (Al, Al_15_Fe_9_Y_2_, and Fe_0.7_Ni_1.3_Al_9_). The more complex crystallization mechanism must occur in the case of the alloy containing Cu, for which four different phases were identified: Al, Al_15_Fe_9_Y_2_, AlNiY, and YCuAl. According to that, the analysis of the formation of the second phase was performed. In the XRD pattern of Al_85_Ni_10_Y_5_ alloy, it can be seen (Fig. 3g) that after the formation of the Al phase as the second one Ni-rich Al_19_Ni_5_Y_3_ phase crystallizes. This phase crystallization was observed at 613 K. At a much higher temperature (673 K), crystallization of the Al_15_Fe_9_Y_2_ phase was noted for the alloy with both, Ni and Fe (Fig. 3h). Interestingly, for Al_85_(Ni, Fe, Cu)_10_Y_5_ alloy, the spontaneous formation of two different phases was observed. The Al_15_Fe_9_Y_2_ phase crystallizes with the AlNiY phase leaving a copper-rich residual amorphous phase (Fig. 3i). This residual amorphous phase crystallizes in the last stage into YCuAl, whereas the crystallization of AlNiY and Fe_0.7_Ni_1.3_Al_9_ was related to the last crystallization stage of the Al_85_Ni_10_Y_5_ and Al_85_(Ni, Fe)_10_Y_5_ alloys, respectively.

The crystallization process for all the alloys is schematically shown in Eqs. (1–3). Generally, the first crystallization stage is the same for all alloys and is related to the precipitation and growth of Al nanocrystals. Afterwards, intermetallic phases crystallize from the TMs rich residual amorphous matrix. The crystallization of these phases is also not accidental. Firstly, crystalize intermetallic phases rich in the aluminium such as Al_19_Ni_5_Y_3_ and Al_15_Fe_9_Y_2_. Finally, the residual amorphous phase crystallizes in intermetallic phases rich in TMs such as AlNiY and YCuAl.

1 $$\begin{aligned} {\text{amorph}}_{{{\text{Al85Ni1}}0{\text{Y5}}}} & \to {\text{Al}} + \left( {{\text{amorph}}} \right)_{{{\text{TM}} - {\text{rich}}}} \to {\text{ Al }} + {\text{ Al}}_{{{19}}} {\text{Ni}}_{{5}} {\text{Y}}_{{3}} + \, \left( {{\text{amorph}}} \right)_{{{\text{resid}}}} \\ & \to {\text{ Al }} + {\text{ Al}}_{{{19}}} {\text{Ni}}_{{5}} {\text{Y}}_{{3}} + {\text{AlNiY}} \\ \end{aligned}$$

2 $$\begin{aligned} {\text{amorph}}_{{{\text{Al85}}\left( {{\text{Ni}},{\text{Fe}}} \right){1}0{\text{Y5}}}} & \to {\text{Al}} + \left( {{\text{amorph}}} \right)_{{{\text{TM}} - {\text{rich}}}} \to {\text{ Al }} + {\text{ Al}}_{{{15}}} {\text{Fe}}_{{9}} {\text{Y}}_{{2}} + \, \left( {{\text{amorph}}} \right)_{{{\text{resid}},{\text{ Ni}} - {\text{rich}}}} \\ & \to {\text{ Al }} + {\text{ Al}}_{{{15}}} {\text{Fe}}_{{9}} {\text{Y}}_{{2}} + {\text{ Fe}}_{{0.{7}}} {\text{Ni}}_{{{1}.{3}}} {\text{Al}}_{{9}} \\ \end{aligned}$$

3 $$\begin{aligned} {\text{amorph}}_{{{\text{Al85}}\left( {{\text{Ni}},{\text{Fe}},{\text{Cu}}} \right){1}0{\text{Y5}}}} & \to {\text{Al}} + \left( {{\text{amorph}}} \right)_{{{\text{TM}} - {\text{rich}}}} \to {\text{ Al }} + {\text{ Al}}_{{{15}}} {\text{Fe}}_{{9}} {\text{Y}}_{{2}} + {\text{ AlNiY }} + \, \left( {{\text{amorph}}} \right)_{{{\text{resid}},{\text{ Cu}} - {\text{rich}}}} \\ & \to {\text{ Al }} + {\text{ Al}}_{{{15}}} {\text{Fe}}_{{9}} {\text{Y}}_{{2}} + {\text{ AlNiY }} + {\text{YCuAl}} \\ \end{aligned}$$

**Figure 4:**
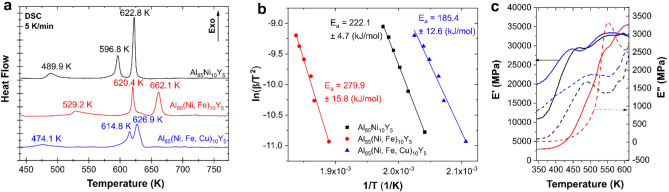
(**a**) Comparison of DSC curves recorded at the heating rate of 5 K/min for Al_85_TMs_10_Y_5_ amorphous alloys, (**b**) Kissinger’s plots for the first observed in DSC curves peak related to the crystallization of Al phase with marked calculated activation energies, (**c**) DMA curves: storage (*E′*) and loss (*E″*) modulus as a function of temperature recorded for Al_85_TMs_10_Y_5_ amorphous alloys (the colors correspond to the legend in graphics **a** and **b**).

The proposed crystallization mechanism was compared with DSC and DMA measurements. The analysis of DSC curves can provide information about the crystallization onset, the number of crystallization stages, and the activation energy of crystallites formation and growth^36,38^. A few-stage crystallization process was observed under the analysis of DSC curves (Fig. 4a). According to the analysis of in-situ high-temperature XRD patterns, the obtained DSC curves are consistent with the developed crystallization mechanism. Moreover, for Al_85_(Ni, Fe, Cu)_10_Y_5_ amorphous alloy was confirmed that it is possible to crystallize two different phases (Al_15_Fe_9_Y_2_ and AlNiY) at the second crystallization stage, which manifest itself with two overlapping peaks. In the case of the remaining alloys, all crystallizations occur separately at different temperatures. To better understand the crystallization process of the first phase from the amorphous matrix, the average activation energies (*E*_*a*_) were calculated using the Kissinger model for non-isothermal crystallization^39^. The *E*_*a*_ obtained from Kissinger’s plots for the first crystallization peak at a heating rate of 5–30 K/min (Fig. 4b) are consistent with the thermal stability of the amorphous phase. According to the calculations, the activation energy of crystallization of Al for Al_85_Ni_10_Y_5_ alloy is equal to 222.1 ± 4.7 kJ/mol and 279.9 ± 15.8 kJ/mol for Al_85_(Ni, Fe)_10_Y_5_ alloy. The Al_85_(Ni, Fe, Cu)_10_Y_5_ alloy is characterized by the lowest *E*_*a*_ value equal to 185.4 ± 12.6 kJ/mol, which explains the ultrafast crystallization of Al phase observed in *situ* high-temperature XRD measurements. Obtained herein values of *E*_*a*_ are much higher than observed, for example, for Al_89_La_6_Ni_5_ amorphous alloy, for which activation energy for precipitation of Al nanocrystals determined using Kissinger model was equal to 169 ± 10 kJ/mol^34^. Moreover, Al_85_Ni_10_Y_5_ and Al_85_(Ni, Fe)_10_Y_5_ alloys have activation energy much higher than Al_86_Ni_6_Y_6_Ce_2_ alloy (186.00 ± 14.78)^40^. Accordingly, all tested Al_85_TMs_10_Y_5_ amorphous alloys are characterized by relatively high thermal stability.

DMA technique was recently applied to determine the glass transition temperature and crystallization process of amorphous alloys, especially Zr-based ones^41^. In this study, the DMA technique was used to compare the mechanical properties with the crystallization of different phases. Sometimes analysis of DMA curves can provide information about the α and β mechanical relaxations in amorphous metallic alloys. These analyses are generally used for the Zr-based alloys; however, some interesting findings were also presented for Fe-based alloys^42^. Herein the storage (*E′*) and loss (*E″*) modulus as a temperature function were measured and are presented for all amorphous samples in Fig. 4c. The presence of glass transition should be related to the drop of the *E′* value and peak on the corresponding *E″* curve, which was not observed for any analyzed samples. Observed herein, the spontaneous growth of both values corresponds to the crystallization process, which results in the precipitate-hardening of Al-based amorphous alloys. According to that, when the crystallization process occurs, the storage modulus increases, and maximum on the *E″* can be observed. The behavior observed on these curves is consistent with other measurements. The crystallization process of Al_85_(Ni, Fe, Cu)_10_Y_5_ alloy starts very fast, which can be related to the presence of the most ordered structure in comparison to other samples (the *E′* at 350 K is near two times higher than for Al_85_(Ni, Fe)_10_Y_5_ and near ten times higher than Al_85_(Ni, Fe)_10_Y_5_ alloy). Also, the crystallization of the other phases can be visible. The Al-TM-Y intermetallic phases’ growth occurs, which manifests itself by the second growth of the *E’* value and the presence of the second peak at *E″*. However, the hardening of these Al-based alloys by the intermetallic phases is too high, and samples become too brittle for further testing and observation of crystallization of the last phases.

## Conclusions

The Al_85_TMs_10_Y_5_ alloys with Ni, Fe, Cu, and Cr chemical elements as the transition metals influenced the glass-forming ability were successfully prepared using the melt spinning method. The negative impact of the Cr addition on the GFA was observed and described for analyzed alloying system. Moreover, performed in this study analysis of the influence of TMs on the glass-forming ability of Al_85_TMs_10_Y_5_ alloys allowed to state that the most thermal stable amorphous structure can be obtained for Al_85_(Ni, Fe)_10_Y_5_ alloy, whereas the Al_85_(Ni, Fe, Cu)_10_Y_5_ alloy can be characterized by the ultrafast crystallization process. Interestingly, the presence of Cu and the precipitation of the YCr_2_Al_20_ phase results in the highest corrosion resistance compared to homogeneous amorphous alloys. Furthermore, the anticorrosion properties of Al_85_(Ni, Fe)_10_Y_5_ alloy is much higher than for other amorphous samples. The crystallization mechanisms of amorphous alloys based on in-situ high-temperature X-ray diffraction studies, consistent with the measurement data obtained from DSC and DMA experiments, was described and disccused. Accordingly, there is a possibility to use this technique to show the amorphous structure behavior at different temperature ranges and determine the processing parameters (time and temperature), which could precipitate Al crystals in the amorphous matrix without any additional studies.

## Data Availability

The data and material generated during and/or analyzed during the current study are available from the corresponding author on reasonable request.
